# Robot-Assisted Radical Prostatectomy: A Step-by-Step Guide

**DOI:** 10.1089/end.2017.0723

**Published:** 2018-05-01

**Authors:** Linda M. Huynh, Thomas E. Ahlering

**Affiliations:** Department of Urology, University of California Irvine Health, Orange, California.

**Keywords:** robot-assisted radical prostatectomy, radical prostatectomy, daVinci robot, surgical technique

## Abstract

Radical prostatectomy remains an important means to treat prostate cancer. A major limiting factor to radical prostatectomy is short- and long-term complications, especially incontinence and sexual dysfunction. With the advent of robotic radical prostatectomy, the ability to easily evaluate technical issues with video has been realized. In this article, we present a step-by-step examination of our procedure and our results over the past 5 years.

## Indications

Like radical prostatectomy, robot-assisted radical prostatectomy (RARP) is indicated for men with prostate cancer with an acceptable lifetime expectancy.^1,2^ Historically, the primary indication for RARP has been localized disease, but there has been recent evidence that men with nonlocalized disease will likely experience significant improvement in survival and, as such, are an indication as long as a complete discussion of risks, benefits, and complications has been accomplished.^1,2^ Contraindications that may impact the decision for RARP include a history of extensive abdominal or pelvic surgery, morbid obesity, or extremely large prostates. Clinicodemographic information of our most recent patient cohort is presented in Table 1.

**Table 1.** Clinicopathologic and Demographic Information

n* = 459*	*Mean*	*SD*
Age, years	63.4	8.2
Pre-PSA, ng/cc	9.7	14.0
PSA, *n* (%)		
<5	116.0	25
5–10	212.0	46
>10	122.0	27
AUA	9.0	7.2
Bother	1.8	1.5
IIEF-5, *n* (%)		
22–25	220.0	48
15–21	127.0	28
Height, lbs	70.4	2.9
Weight, inches	191.9	31.8
BMI	27.1	3.9
EBL	87.5	33.5
Prost Wt, g	54.9	21.1
pGS, *n* (%)		
≤3 + 3	90.0	20
3 + 4	153.0	33
4 + 3	110.0	24
4 + 4	28.0	6
3 + 5	1.0	0
≥4 + 5	61.0	13
P-stage, *n* (%)		
pT2	286.0	62
pT3/T4	161.0	35

AUA = American Urological Association; BMI = body mass index; EBL = estimated blood loss; pGS = pathologic Gleason score; PSA = prostate-specific antigen; SD = standard deviation; IIEF-5 = international index of erectile function.

## Preoperative Preparation

Before surgery, it is our routine to encourage significant lifestyle goals. As such, we counsel patients on the benefit of heart healthy dietary habits, exercise, nonsmoking, and (if indicated) weight loss. With specific reference to RARP, we systematically and strongly encourage preoperative Kegel exercises, three times a day. If sedentary, we also strongly recommend walking 1 to 2 miles daily in preparation for surgery. A few days before the surgery date, we also recommend nightly, low-dose phosphodiesterase (PDE-5) inhibitors (5 mg tadalafil or 20 mg sildenafil). Before surgery, we also check total testosterone and sex hormone binding globulin (SHBG) levels, and calculate free testosterone levels.

We do not have any standing rules on a “must-wait” period of 6 weeks from the date of prostate biopsy and surgery. We also do not use preoperative bowel preparations. In young patients who might consider fathering children, we do recommend sperm preservation.

## Patient Positioning

**FIG. 1.**
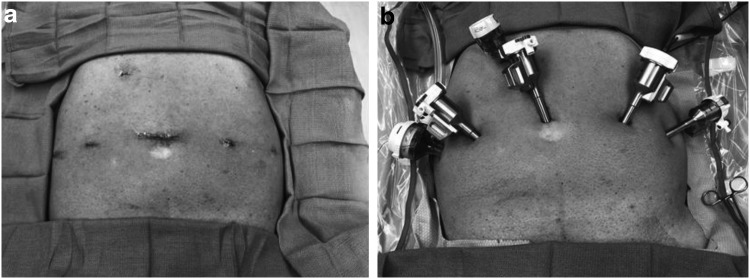
Patient positioning and port placement. (a) Port placement. (b) Port positioning.

All procedures are performed under general anesthesia. The patient is positioned securely on the operating table with stirrups, prepped, and draped (Fig. 1). We believe it is important to have flexure at the hips to reduce the risk of stretch injury either to the femoral or obturator nerve as they exit the pelvis. After the patient is prepped and draped and a standard “time-out” completed, a Foley catheter is gently placed. Next, a Veress needle is placed in Palmer's point in the left upper quadrant. Once the needle reaches the peritoneal cavity, carbon dioxide is pumped into the abdomen via tubing from an insufflator, creating a pneumoperitoneum at 20 mm Hg. As the pressure slowly rises to 20, the port sites are prepared. Once at 20 mm Hg, the first port, the camera port is placed through a transverse incision just above the navel followed by the remaining five ports all under direct vision. Once all ports are positioned, AirSeal is installed and activated and the pneumoperitoneum is reduced to 12 mm Hg for the procedure.

Men are then positioned in the Trendelenburg position, allowing gravity to gently pull the abdominal contents out of the pelvis, facilitating access to the bladder and prostate, and reducing the risk of injury to abdominal organs. The legs are separated to facilitate docking of the daVinci robot. Once the patient is positioned, the robot is docked. Figure 1 depicts patient positioning.

## Surgical Steps

### Releasing the bladder

The bladder is released by incising the peritoneum from the right obliterated umbilical artery to the left obliterated umbilical artery and then down to the vas deferens and then deep into the perirectal space (Supplementary Video S1; Supplementary Data are available online at www.liebertpub.com/end). This facilitates retraction of the bladder and rectum out of the pelvis to improve space. The anterior prostatic fat (APF) is dissected to skeletonize the puboprostatic ligaments for optimal visualization of the apex. It is further dissected off of the anterior prostatic capsule from the apex to the bladder neck. This step helps visualize the border between the prostate and bladder neck. In addition, it is sent for a pathologic examination in case the pathologist reports a positive surgical margin (PSM). If a PSM is seen anteriorly, the APF can be evaluated for residual cancer, which in our experience has always been negative. We additionally published that about 15% of men will have lymph nodes in the APF and that in 2% to 3% of men, this will be the only site of metastasis.

### Endopelvic fascia

The endopelvic fascia is initially incised laterally closer to muscle than the nerve. We do this to fully expose the neurovascular bundles (NVBs) to facilitate visualization and reduce traction injury. In the Supplementary Video S2, two somatic nerves are seen and preserved. Continuous retraction of the prostate is key to safe and speedy progress. At the apex, we completely release Myer's muscle to visualize and protect the apex, NVB, and urethra when transecting the dorsal venous complex (DVC) and remaining apical structures. It is important to fully visualize the distal posterior apex as demonstrated by the arrow. We liberally irrigate bleeders with cold sterile water (at 4°C) to precisely visualize and cauterize them, which has been shown to essentially eliminate thermal spread and injury.

### Anterior and posterior bladder neck

The site of transection of the anterior bladder neck is visually facilitated by retracting the Foley balloon (Supplementary Video S3). Cautery should be set at ∼25 W. Once clearly into muscle, the dissection is performed largely without cautery to better differentiate muscle, prostate, and urethra. Cautery is used to open the urethra with good hemostasis. Transection of the bladder from the prostate is facilitated with retraction of the left hand with continuous sufficient force to maximize visualization. Once the bladder is entered, we prefer to retract the prostate with fourth arm rather than use the catheter. This facilitates a quick method to change angles and improve visualization and dissection planes.

The interior of the bladder is inspected logistically. It is preferable to grab the prostate with the fourth arm as shown at the 6 o'clock position. The intent is to enter the muscular/vascular space behind the detrusor (or the posterior bladder neck). This will preserve the full thickness of the posterior bladder as it is transected from right to left. Immediately behind the posterior bladder are the longitudinal muscle and multiple vessels that need cauterization. This muscular/vascular layer is later incorporated into the Rocco stitch.

### Seminal vesicles and rectum

An important potential advantage of this approach to the posterior approach is that the hypogastric nerves innervating the seminal vesicles (SVs) and the bladder neck are not transected (Supplementary Video S4). In our center's experience, the key to mobilization of the SVs is to focus on lifting the SVs with minimal traction to the surrounding hypogastric nerves, which are important for the sensation of orgasm. The SVs are then used to lift the prostate for separation from the rectum. Denonvilliers is grasped and lifted and incised sharply until the perirectal fat is seen. Dissection of the plane between the prostate and rectum is facilitated by the surgeon's left hand elevating the prostate as the assistant retracts gently but firmly on the rectum with the sucker as needed. The dissection is carried distally to the apex.

### “Clipless” transection of the prostatic pedicles and the NVBs

This approach to the prostatic pedicles' nerve bundles focuses on minimizing traction—hence no use of clips (Supplementary Video S5). We prefer to suture ligate the pedicles with 3-0 V-lock sutures. For thick pedicles as in this case, touch cautery at 35 W is preferred. Critical to nerve preservation is holding the prostate with the fourth arm, sharply releasing the nerve from the prostate without traction. Cauterization is optimized by using one blade of the open scissors. Thermal injury results from desiccating bipolar cauterization. Touch cautery (that is cut and cauterize) has no measurable thermal injury. In a study of 880 consecutive cases, we found that after adjusting for important patient parameters, touch cautery and the athermal technique had similar potency outcomes. In this study, we demonstrate how the prostate is lifted and sharply freed from the nerve with minimal traction of the nerve. We also recommend that the distal half of the left nerve dissection be performed “left handed.” Again the prostate is lifted and rotated to facilitate sharp dissection and minimal traction or touching of the nerve.

### DVC and urethral transection

In 2012, we stopped using a stapler as our new nurse practitioner was too small to manipulate the stapler appropriately (Supplementary Video S6). So abruptly in September 2012, we switched from the stapler to cutting the dorsal vein and urethra and then oversewing the DVC. Within 6–12 months we noted dramatic improvement in both continence and early return of erections.

The pad-free continence rate at 30 days jumped from 43% to 54% and the 1-year pad-free rate for the different age groups from 40 to 80 years of 100%–94%, respectively (Table 2). We also saw improvement at 3 months in the proportion of potent men (defined as an international index of erectile function-5 [IIEF-5] >17) jump from 30% to 60%. With this technique, one is able to pull and fully rotate the prostate, facilitating circumferential release and maximizing urethral length without tracking on the nerves. The prostate is rotated to visualize the posterior capsule, which guides the dissection. At the apex, spreading the scissor tips allows the surgeon to sense and enter open space between the large veins. Where risk of a margin exists we simply transect further away from the prostate. In our experience, maximizing urethral length appears to improve early continence via improved sensation. We oversew the DVC with a 4-0 V-lock suture in all cases to prevent late or delayed venous bleeding. Arterial bleeding along the nerves sutured with 4-0 vicryl. The prostatic pedicles are oversewn with a 3-0 V-lock suture for arterial but more importantly venous hemostatic security. We believe delayed venous bleeds come from large veins in the bladder neck near the pedicles.

**Table 2.** Thirty-Day and Overall Continence Recovery, by Age Group

	*30-day continence*			*Overall continence*		
	*Continence*	n	*%*	*Continence*	n	*%*
<65	127	198	64.1	192	198	97.0
65–74	60	120	50.0	114	120	95.0
>74	7	16	43.8	15	16	93.8
All	188	345	54.5	321	334	96.1

### Rocco and Van Velthoven anastomosis

**FIG. 2.**
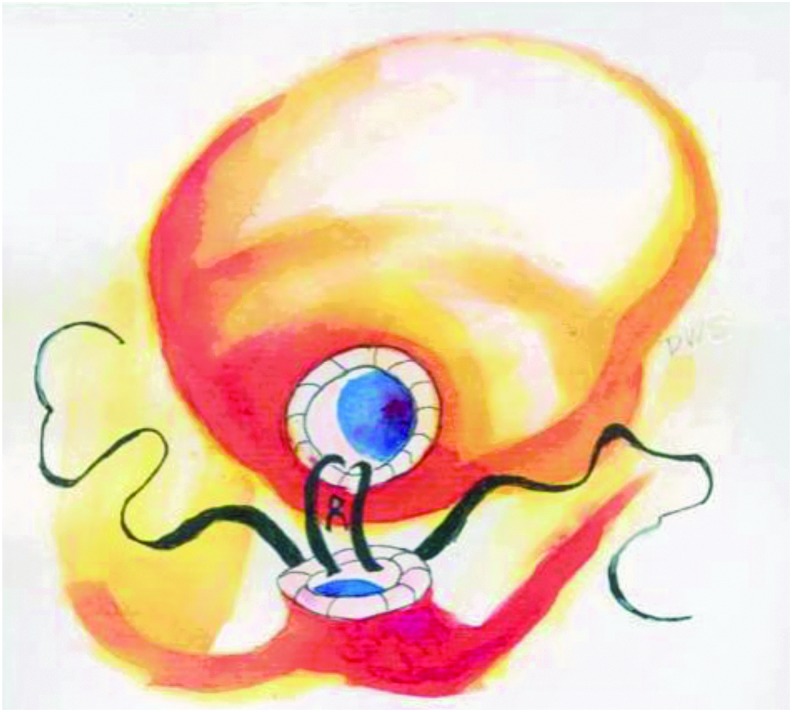
Single-knot method for laparoscopic running urethrovesical anastomosis.

We utilized the Rocco stitch as we believe it simplifies anastomosis and significantly reduces postoperative hematuria and reduces bladder neck contractures (Supplementary Video S7). A 3-0 V-lock begins at the bladder incorporating the cut edge of Denonvilliers and then the posterior bladder detrusor. The next suture is intended to incorporate as much of the muscular structural support behind the urethra as possible. We stress that only the bladder is pulled toward the urethra to avoid pulling and tearing out from the urethral side. The anastomosis is the standard single-knot Van Velthoven anastomosis (Fig. 2). We start at 5 o'clock full thickness in the bladder neck throwing six consecutive sutures and then cinching the bladder down to the urethra. The suture continues up to 10 o'clock. The second suture runs up the other side.

## Postoperative Care

Beginning the first day after surgery, an early ambulation protocol is followed for all patients. We stress sitting upright in a chair and walking as much as tolerated. We counsel patients to walk 0.5 mile on postoperative day 1 and 1 mile or more the following days until the catheter is removed. A total of 97%–98% of patients are discharged from the hospital the morning of postoperative day 1. All patients are discharged with a urinary catheter in place. Two urine collection bags are provided to facilitate day and nightly wear. The catheter is generally removed after 6 to 7 days. Cystograms are rarely indicated and only in the event of persistent gross hematuria. After catheter removal, patients are asked to track their daily urinary pad use until 3 days of pad-free status returns, which will be documented by mailing in a “pad-free” postcard or daily urinary pad log confirming continence.^3,4^ In addition, if after the first week postcatheter removal, a patient is utilizing two or more “wet” urinary pads, an anticholinergic medication for 2 to 3 months is recommended to reduce the time to pad-free status.^5^

Regarding sexual function recovery, patients are advised to take a daily dose of a PDE-5 inhibitor (preferably at nighttime). Patients are advised that sexual function recovery may occur over 1 to 2 years post-RARP. Our surgical technique regarding accessory pudendal arteries (APAs) was recently published.^6^ While surgical preservation of APAs is optimal, we presented data from a robust patient cohort that sacrifice of APA(s) during RARP did not lessen recovery of erectile function. We found no evidence that transecting one or more APA(s) reduced recovery of erections or potency in normal baseline function of men. This was also true in patients with mild-to-severe preoperative erectile dysfunction (ED) and/or advanced age. To predict men who are at the highest risk for long-term (2+ years) erectile dysfunction, studies have suggested adopting a percent erection fullness scale as an adjunct to the erections sufficient for intercourse and IIEF-5 questionnaires. These studies suggest that men who report <25% fullness at 90 days post-RARP may benefit most from secondary interventions for erectile dysfunction.^7,8^ In contrast, men reporting >24% fullness are likely to recover sexual function 2 years following radical prostatectomy.

## Troubleshooting

### Common perioperative complications

In their systematic analysis, Novara et al.^1^ found that the most common complications following RARP are lymphocele/lymphorrhea (3.1%), urine leak (1.8%), and reoperation (1.6%). Mean in-hospital stay was 1.9 days post-RARP. Detection on ultrasound or computed tomography imaging is facile, and although most lymphoceles are self-resolving, if needed drainage via ultrasonic guidance may be used.^1^ In a study by Coelho et al.,^9^ anastomotic leakage varied from 4% during the learning curve to 0.3% in later experience, demonstrating the importance of surgeon experience for minimizing complications. Finally, a recent analysis of the National Surgical Quality Improvement Program (NSQIP) database showed reoperation rates were significantly lower for patients undergoing radical prostatectomy via minimally invasive techniques (including robot-assisted and laparoscopic techniques) compared with open radical prostatectomy (ORP) (1.1% *vs* 1.5%). Bleeding (0.09% of all cases and 20.83% of reoperated cases), wound dehiscence (0.06% of all cases and 13.9% of reoperated cases), and urinary retention (0.05% of all cases and 11.1% of reoperated cases) were the most common reasons for reoperation.^10^

### Prior transurethral resection of the prostate

A history of transurethral resection of the prostate may be more difficult to perform particularly for less experienced surgeons. The reason is that the normal anatomy of the bladder neck may frequently be quite distorted. The distortion makes it more of a problem to assess where the prostate ends. In addition, the bladder neck opening is significantly bigger and will usually need surgical reconstruction. We recommend plication of the bladder neck at the 3 and 9 o'clock positions. We recommend against a 6 o'clock position as this posterior position has the greatest amount of tension and the crossing of two suture lines increases the risk of distraction and urinary leakage.

### Hernia repair

Some patients have pre-existing inguinal hernias that should be fixed at the time of RARP. Occult hernias are usually only detected at surgery. If a hernia is not corrected, there is significant evidence it will become symptomatic and will need to be repaired, usually within the first year or two. While it is not entirely clear why these hernias occurs, it has been well documented that following standard radical prostatectomy (RP), the risk of needing a hernia repair approaches 10%–20%.^11^ Risk factors for hernias after surgery include failure to fix an occult hernia, a history of hernia repair, smoking, and possibly anastomotic stricture.

**FIG. 3.**
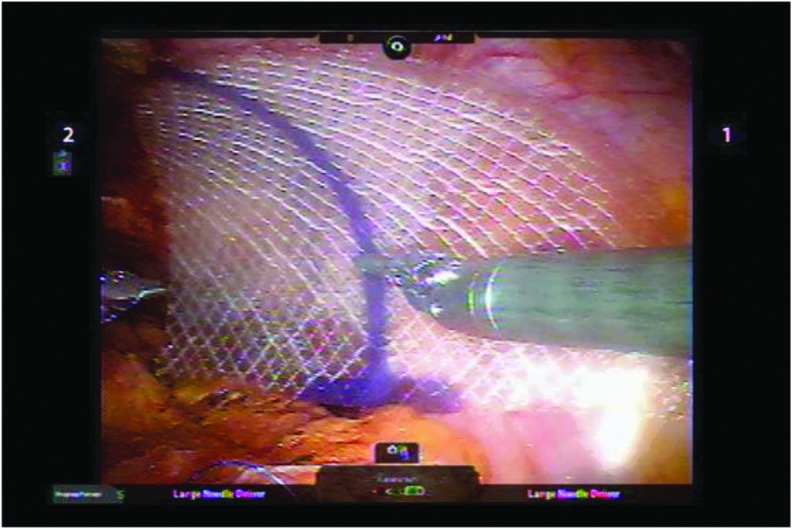
Inguinal hernia with applied flat mesh, secured to Cooper's ligament inferiorly and along the superolateral borders to the rectus sheath.

At the time of RARP, we routinely repair hernias. This is accomplished by securing a 3″ × 4″ piece of sterile synthetic material (our institution carries Proceed mesh) over the inguinal ring (Fig. 3). The addition of this procedure adds only about 10 minutes of operative time. Our group published the first two series of inguinal hernia repair during RARP showing it is safe and easy. In our last 700 patients, we found 134 men with hernias (19%) that were repaired. Only one has subsequently recurred (1.4%).

Incisional hernias also occur and the risk has been well documented and appears to be ∼5%. These incisional hernias essentially always occur through “vertical” camera/extraction port sites. The risk of developing these hernias can be reduced to well below 1% simply by making the incision transverse (our citation). In the last 1000 transverse incisions, the risk has been reduced to ∼0.5%.

## Conclusions

RARP has shown to be an easily acquired laparoscopic technique, with shorter learning curves that rival the open procedure as best practice. We have witnessed a paradigm shift from open to robotic radical prostatectomy as the procedure of choice worldwide. When compared with the open approach, early studies indicate that robotic prostatectomy has equal outcomes in short-term oncologic control, continence, and potency with potentially favorable perioperative outcomes such as in blood loss and transfusion rates, minor complications, narcotic use, convalescence, and length of hospital stay. Initial long-term oncologic and quality-of-life outcomes have also demonstrated similar outcomes to open radical prostatectomy. To view full video, “Full Video – RARP by TA.mp4,” click here. Descriptive video captions are available online in Supplementary Data.

## Recommended Videos from Videourology

1. Kumar A, Samavedi S, Mouraviev V, Marquinez J, Patel VR. Tips and Tricks to Improve Neurovascular Bundles Preservation During Robot-Assisted Radical Prostatectomy. January 2017, 31. https://doi.org/10.1089/vid.2016.0042
2. Abdel Raheem A, Troya IS, Kim DK, Alabdulaali I, Rha KH. Retzius-Sparing Robot-Assisted Radical Prostatectomy: Step by Step Standardized Surgical Technique. .June 2016, 30. https://doi.org/10.1089/vid.2015.0065
3. Jefferies ER, Koupparis AJ, Gillatt D, Rowe EW. Salvage Radical Assisted Laparoscopic Prostatectomy After Low-Dose Rate Brachytherapy. April 2016, 30. https://doi.org/10.1089/vid.2015.0048
4. Phillips JM, Catarinicchia S, LaRosa FG, Maroni P. Technical Learning Points for Robotic Prostatectomy: Video Assessment of Positive Margins at the Prostate Base in Two Cases. Journal of Endourology Part B, Videourology. November 2012, 26. https://doi.org/10.1089/vid.2012.0025

## Supplementary Material

Supplemental data

Supplemental data

Supplemental data

Supplemental data

Supplemental data

Supplemental data

Supplemental data

Supplemental data

## Abbreviations Used

APAs: accessory pudendal arteries
APF: anterior prostatic fat
BMI: body mass index
NVBs: neurovascular bundles
PSA: prostate-specific antigen
PSM: positive surgical margin
RARP: robot-assisted radical prostatectomy
SD: standard deviation
